# Micro-CT study of male genitalia and reproductive system of the Asian citrus psyllid, *Diaphorina citri* Kuwayama, 1908 (Insecta: Hemiptera, Liviidae)

**DOI:** 10.1371/journal.pone.0202234

**Published:** 2018-08-16

**Authors:** Ignacio Alba-Alejandre, Wayne B. Hunter, Javier Alba-Tercedor

**Affiliations:** 1 Department of Zoology, Faculty of Sciences, University of Granada, Campus de Fuentenueva, Granada, Spain; 2 U.S. Dept. Agriculture, Agricultural Research Service, Fort Pierce, Florida, United States of America; Oklahoma State University, UNITED STATES

## Abstract

The Asian citrus psyllid (ACP), *Diaphorina citri*, is a major vector of the bacteria *Candidatus* Liberibacter asiaticus and *C*.L. americanus, which cause Huanglongbing disease (HLB) (aka Citrus greening disease), considered the most serious bacterial disease of citrus trees. As part of a multidisciplinary project on psyllid biology (www.citrusgreening.org), the results presented here concern a detailed anatomical study of the male reproductive system (testes, seminal vesicles, accessory glands, sperm pump, connecting ducts, and aedeagus) using micro-computed tomography (micro-CT). The study summarizes current knowledge on psyllids male reproductive system and represents significant advances in the knowledge of ACP anatomy.

## Introduction

The Asian citrus psyllid (ACP) *Diaphorina citri* (Hemiptera: Liviidae) was first discovered in Shinchiku (Taiwan) in 1907 [1]. Since then, it has become a major vector of citrus in agriculture, transmitting the bacteria *Candidatus* Liberibacter spp. to citrus crops (e.g. lemons, limes, oranges, grapefruit, tangerines, and kumquats), causing Huanglongbing (HLB), also called citrus greening disease. HLB is considered the most serious disease threatening the citrus industry. HLB causes yield loss as well as small, bitter, unpalatable fruit, and eventually tree death. This pathogen is transmitted to the psyllid vector mainly during nymphal feeding on infected citrus trees [2]. *Diaphorina citri* infected with *C*. Liberibacter reportedly increases in fecundity, producing a greater number of offspring [3]. Today HLB has spread to over 40 different countries in Asia, Oceania, and North as well as South America [4,5].

Studies on psyllid anatomy have been published on: general anatomy [6–11], sperm morphology [12,13], testes structure [14–16], biology on different hosts, effects of temperature [17,18], genetics [19–23], communication between sexes [24], abdominal color, and reproductive potential [25] as well as even daily timing of mating and age at reproductive maturity [26]. Moreover, a preliminary micro-CT anatomical study is available [27]. Although different studies have been conducted specifically on the male reproductive system of different Psylloidea species [28–38], studies on ACP are lacking, with only one classical study of the sperm pump by Schlee using light microscopy to examine different species of Psyllina and Aleyrodina, and briefly commenting on *Diaphorina citri* [39]. Also, Stockton et al. investigated the possible reasons why females prefer mating with orange males over blue males, providing schematics of reproductive systems from dissected specimens [40].

The main aim of this work was to expand the understanding of the ACP male reproductive system, to fill gaps regarding the anatomical morphology. The study presents the first extensive application of the micro-CT techniques on the male reproductive system in psyllids. This is a non-destructive method, which enhances the viewing and understanding of structures in their natural anatomical position, avoiding additional deformation that often occurs during dissections and/or slide preparation.

## Materials and methods

**Fig 1:**
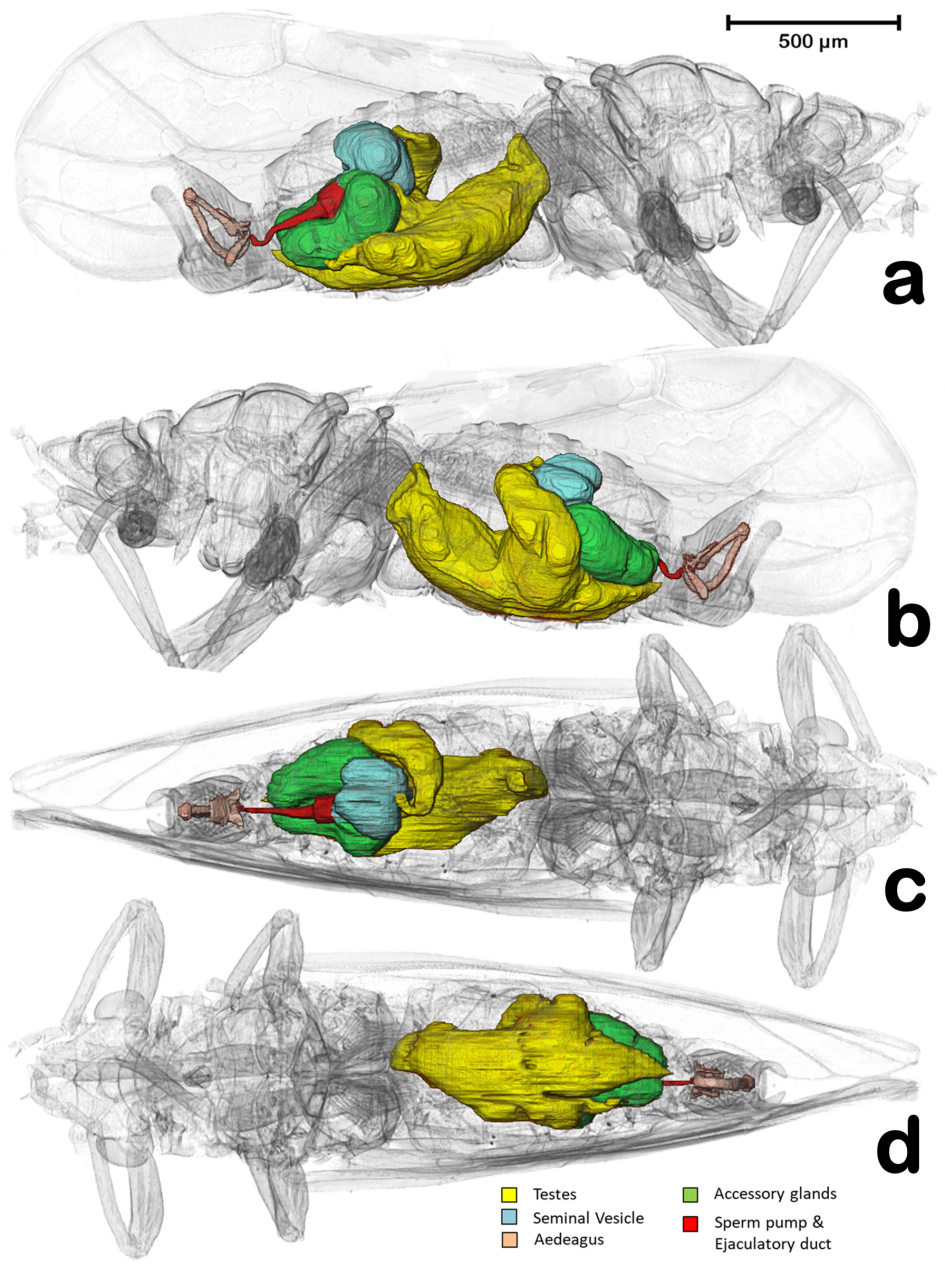
Volume rendering reconstructions of the male reproductive system in its anatomical position, in different perspective views. (**a**) Right lateral, (**b**) Left lateral, (**c**) Dorsal, (**d**) Ventral.

**Fig 2:**
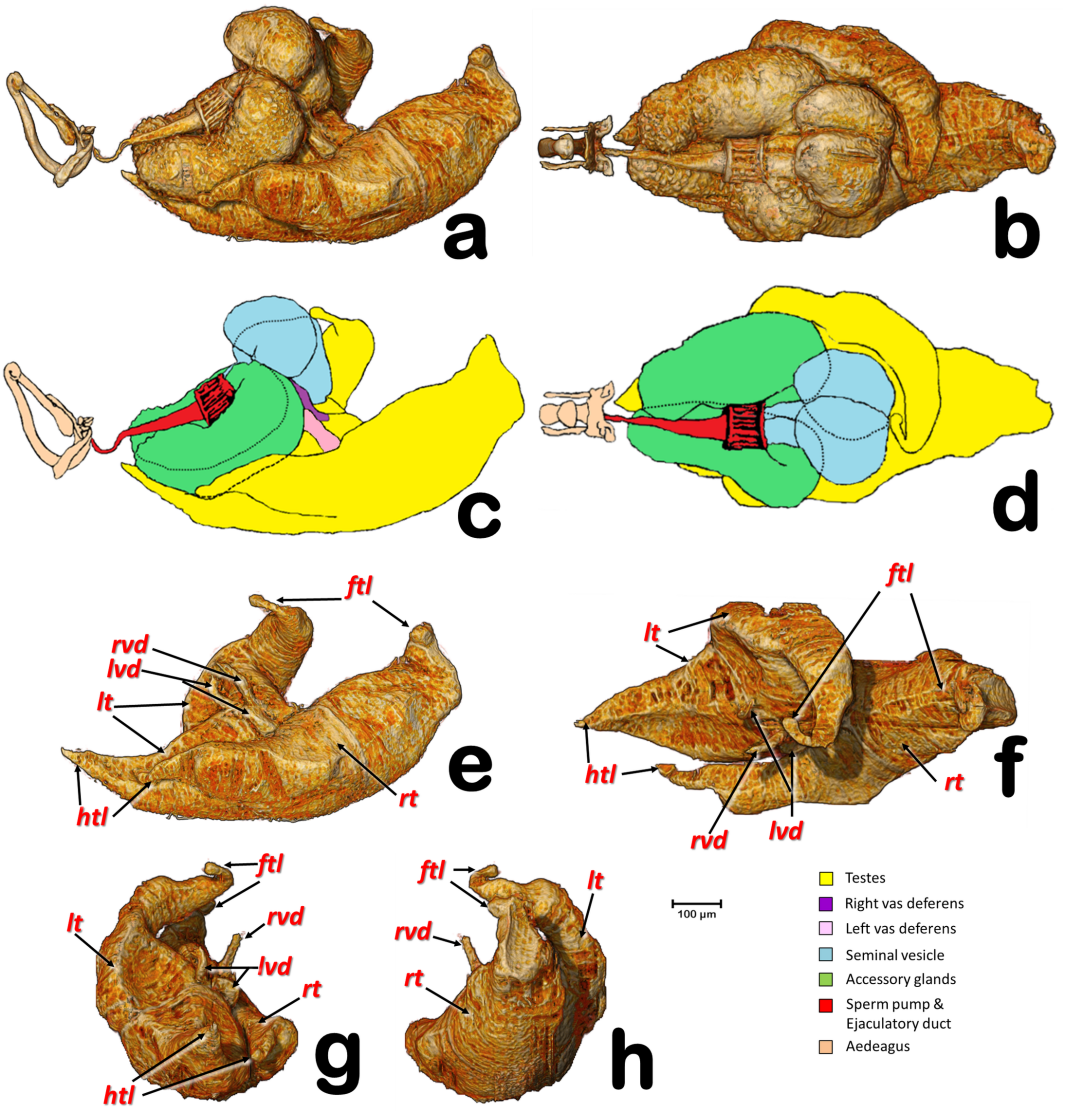
Volume rendering reconstructions of the male reproductive system in different perspective views. (**a, c**) Right lateral; (**b, d**) Dorsal; (**e**) Testes, lateral-; (**f**) Testes, dorsal; (**g**) Testes, postero-anterior; (**h**) Testes, antero-posterior; (**ftl**) Fore testicular lobe; (**htl**) Hind testicular lobe; (**lvd**) Left vas deferens; (**lt**) Left testis; (**rt**) Right testis; (**rvd**) Right vas deferens.

**Fig 3:**
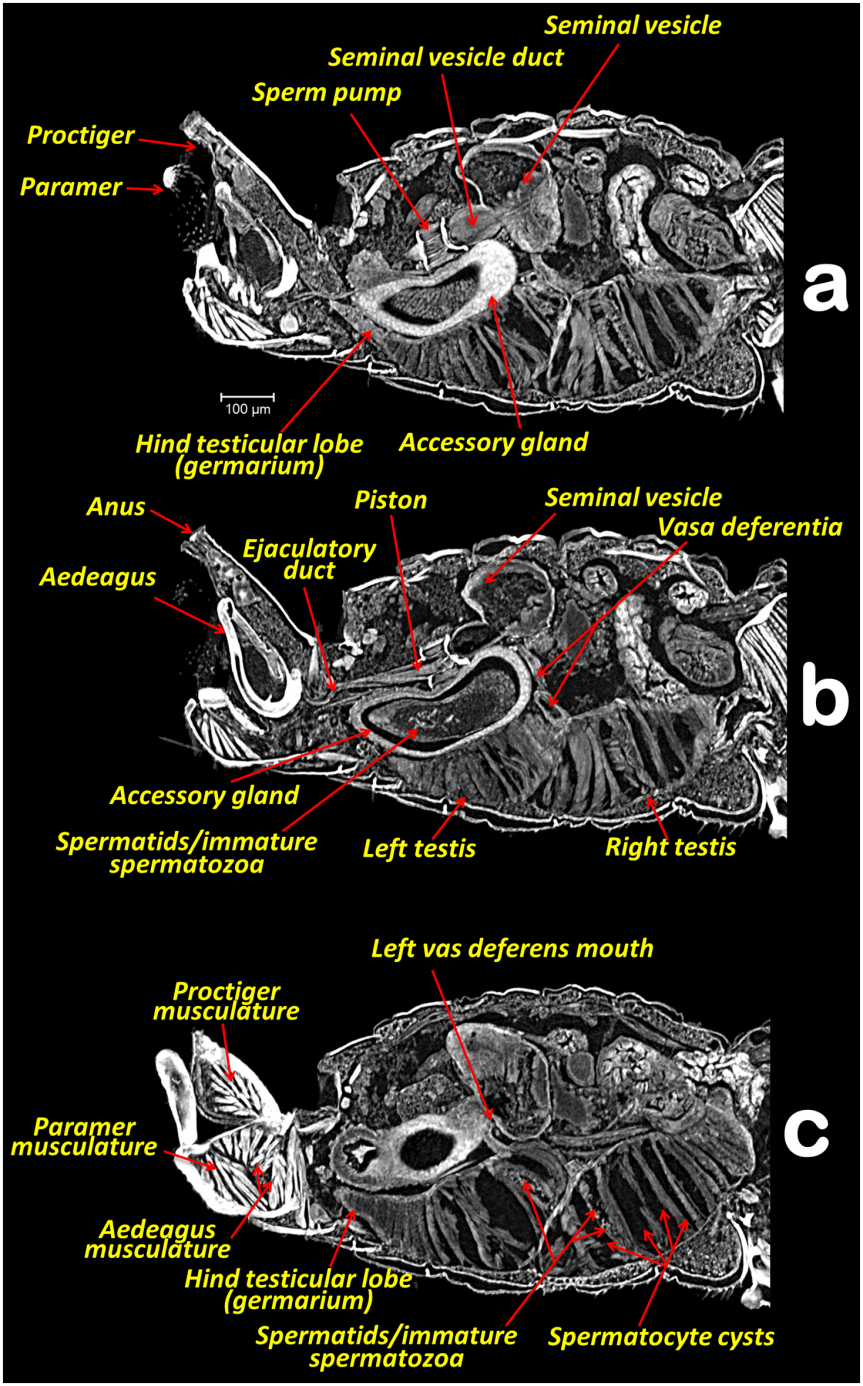
Abdominal middle-left sagittal section views withAmira Multiplanar slicing. (**a**, **b**, **c**) Consecutive slices (23 μm thick) from the right towards the left side.

**Fig 4:**
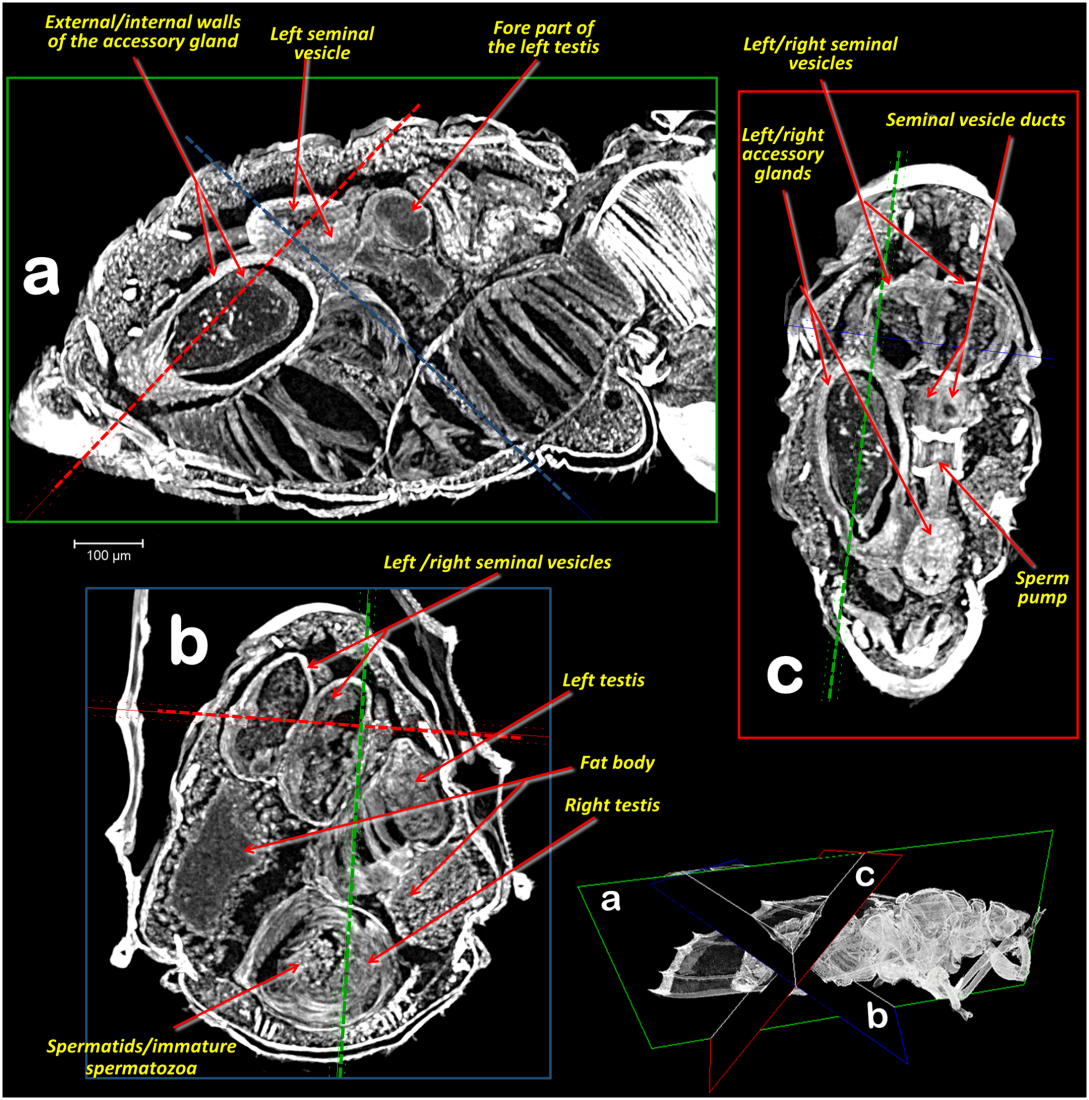
Abdominal Amira Multiplanar sections. (**a**, **b**, **c**) slices (23 μm thick) according to plane views shown in the right-bottom view.

**Fig 5:**
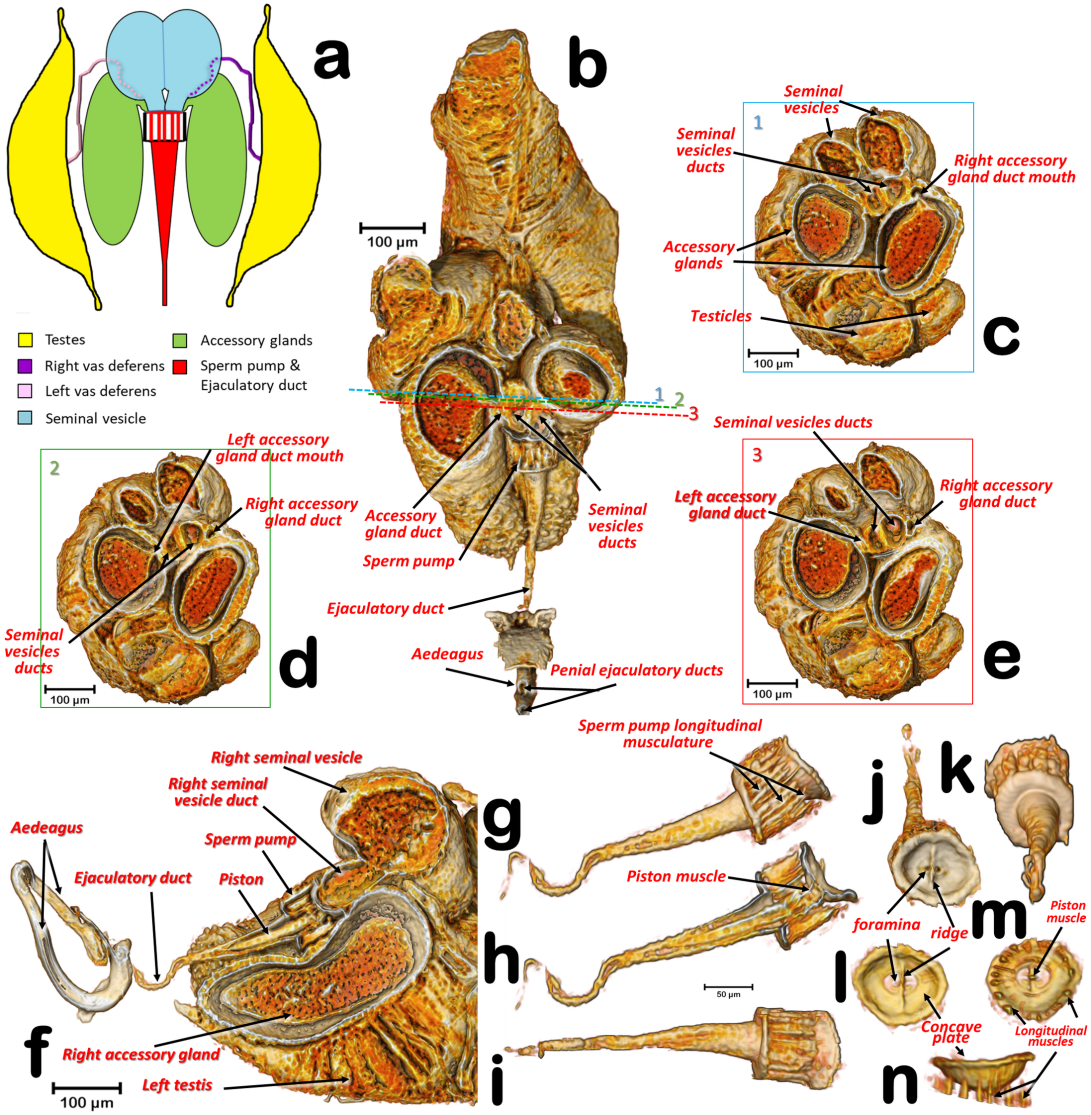
General configuration, sections, and details of the reproductive structures. (**a**) Schematic structure of the male reproductive system, showing the connection between the testes and seminal vesicles (via vasa deferentia) as well as the connection of seminal vesicles and accessory glands to the sperm pump; (**b**) Dorso-ventral (coronal) section at the level of the accessory glands and seminal vesicles ducts. (**c**, **d**, **e**) Antero-posterior (axial) sections of planes shown with blue, green, and red lines numbered as 1, 2, & 3, respectively; (**f**) Sagittal section; (**g**-**k**) sperm pump and ejaculatory duct (**g**: right lateral view, **h**: sagittal section **i**: ventral view, **j**: antero-posterior view, **k**: postero-anterior view); (**l**-**n**) Detail of the anterior plate on the sperm pump showing the foramens **(l**: antero-posterior view, **m**: postero-anterior view, **n**: dorsal view).

**Fig 6:**
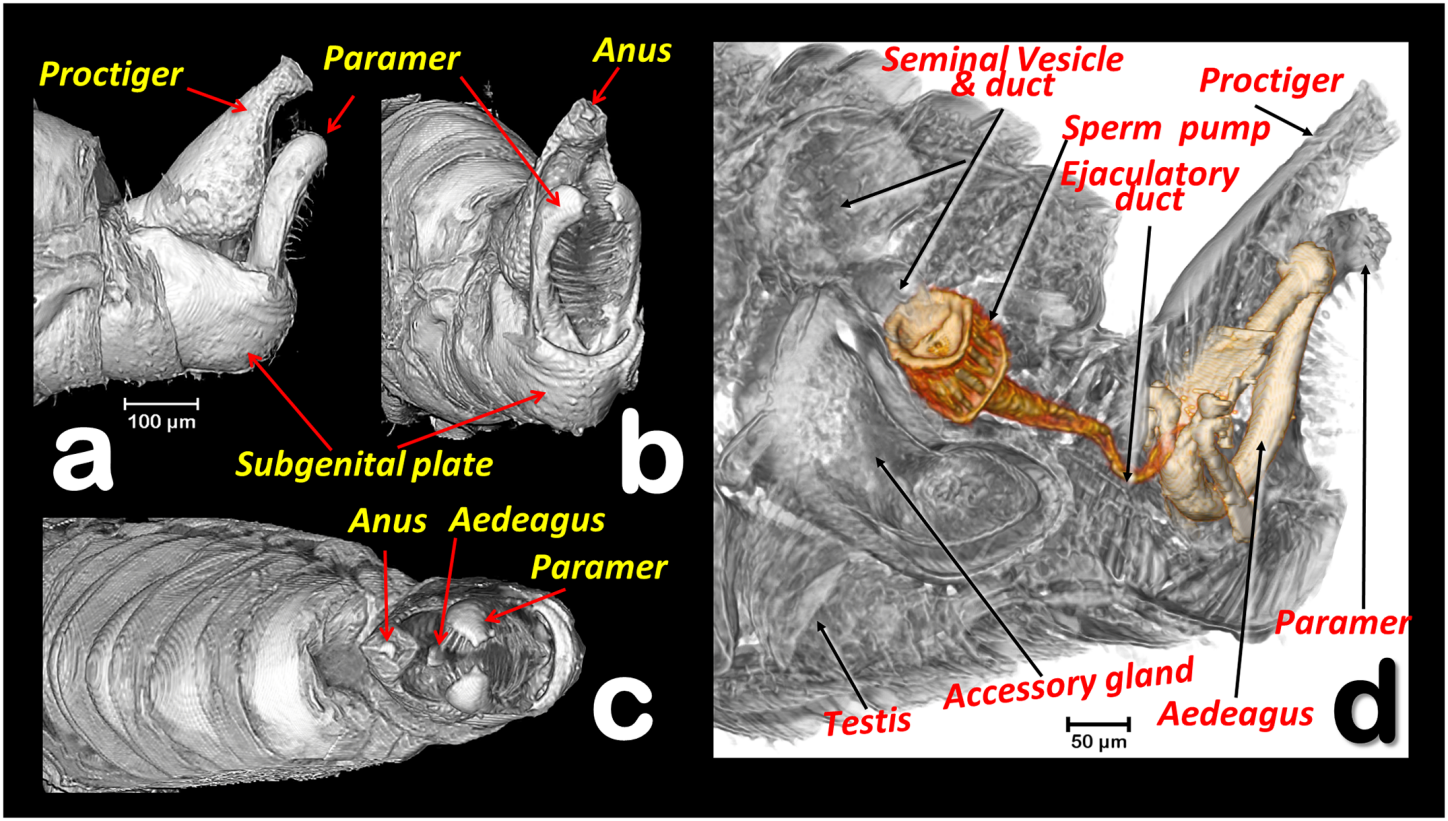
Volume rendering reconstruction of the male abdominal genital terminalia in different perspective views (a-c) and a sagittal right section (d). (**a**) Left lateral, (**b**) Left latero-posterior, (**c**): Apico-dorsal, (**d**) Testis, seminal vesicle, accessory gland, sperm pump, ducts, and aedeagus structures superimposed over a sagittal section of the hind abdominal segments.

**Fig 7:**
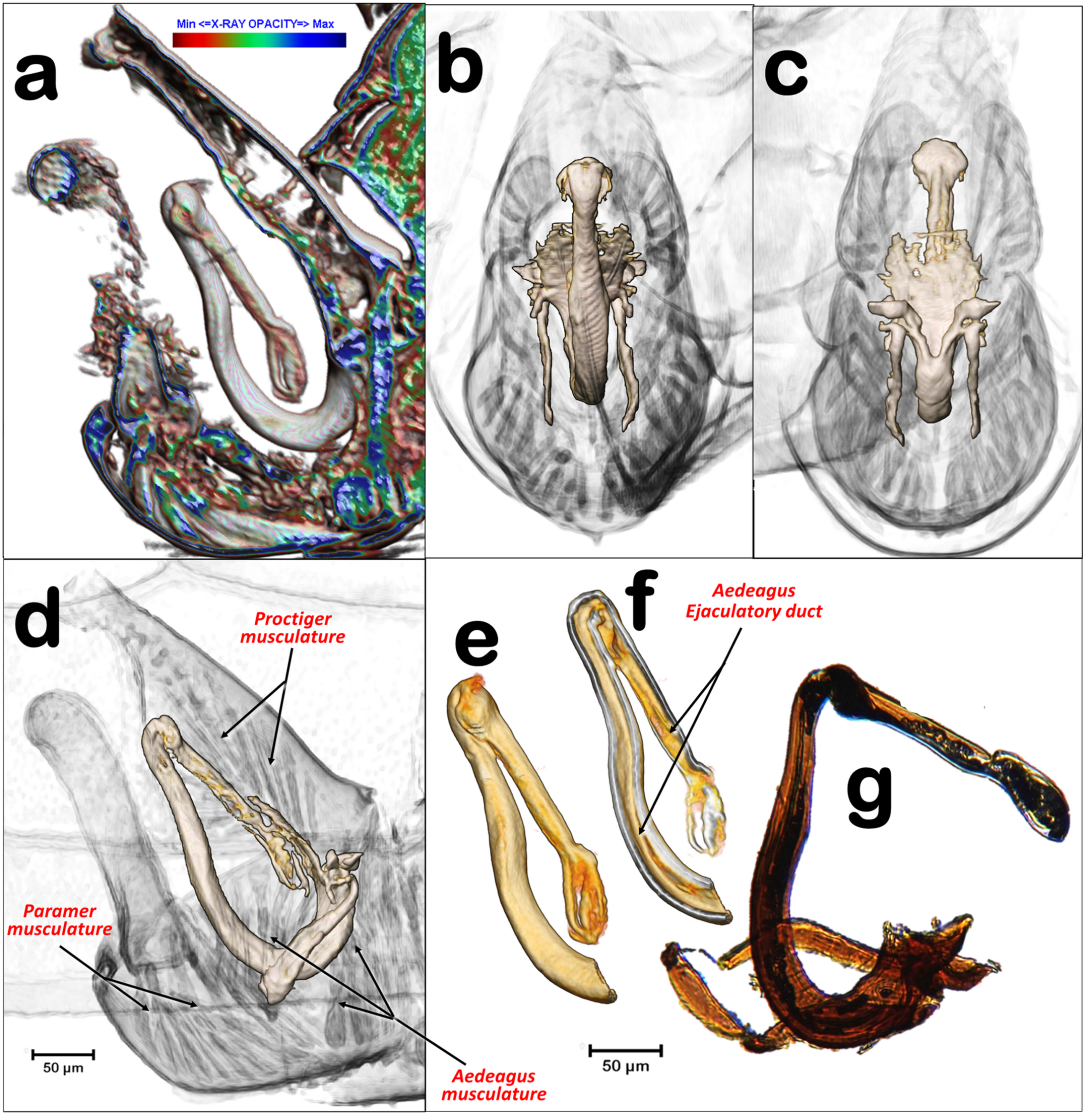
Internal details of the terminalia and aedeagus. (**a**) CTVox volume rendering, (**b-f**) Amira volume renderings, **(g)** light-microscope right lateral view of the aedeagus, mounted in a slide. Volume renderings with details of the male hind abdominal segments showing the anatomical position of the aedeagus structures and associated musculature. (**a**, **d**) right-lateral, (**b**) postero-anterior, (**c**) antero-posterior, transparented to display the sclerotized structures of the aedeagus.

**Fig 8:**
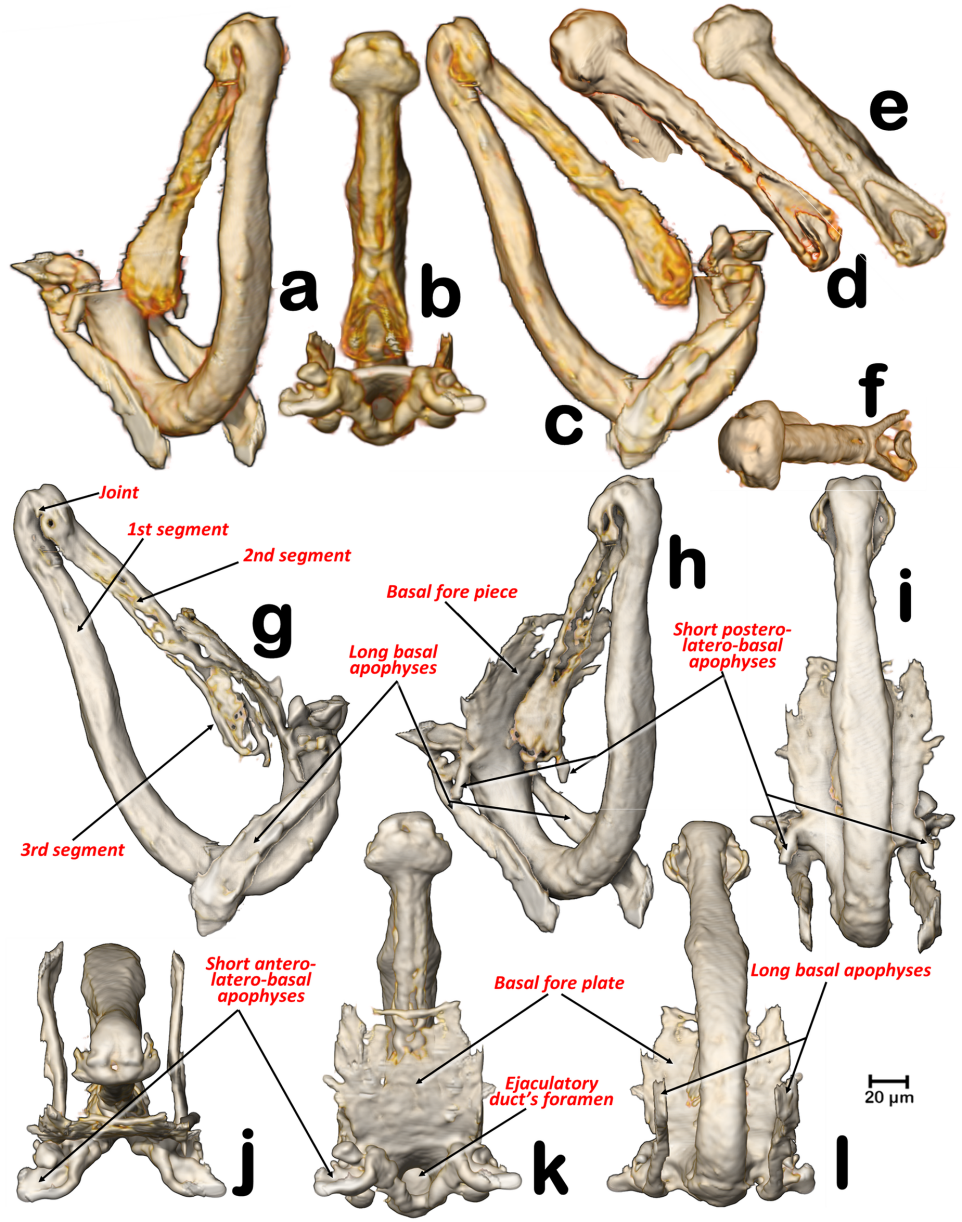
Details of the aedeagus in different perspective views. (**a, h**) angled left-lateral posterior view, (**d**) angled right latero-anterior view, (**b**, **e**, **k**) dorsal, (**c**, **g**) right-lateral, (**i**) postero-anterior, (**f**, **j**) angled dorsal, (**l**) ventral. (**d-l**) For visualization, the renderings have been transparented with Amira software to eliminate soft tissues and enhance harder-sclerotized structures.

The ACP specimens for this study come from the rearing facilities at the United States Department of Agriculture, Agriculture Research Service, Fort Pierce, Florida (USA). Adult specimens shown in Figs 1 to 8 (except that of Fig 7a) were fed for three days on an orange tree sprig submerged in BAPC (***B****ranched* ***A****mphiphilic* ***P****eptide* ***C****apsules*) linked to Hg as a contrast agent [41]. The insects were rinsed three times, 10 min each, with 30% ethanol, dehydrated in an ethanol series (30 min per step, 50%, 70, 80, 90, 95, three times at 100%), and chemically dried by submersion in 2 ml of 100% hexamethyldisilazane (HMDS) for 2 hours, and drying overnight at 35°C. Finally they were glued with cyanoacrylate to the tip of a nylon fishing line 200 μm in diameter, as previously described [7,42]. The prepared insects were scanned with a SkyScan 1172 desktop high-resolution micro-CT, with a Hamamatsu L702 source and a Ximea 11Mp camera. Using the following setting parameters: isotropic voxel size = 0.54μm per pixel; source voltage = 56KV, source current = 43μA, image rotation step = 0.3°, 360° rotation scan, and no filter. The Fig 7a corresponds to a psyllid taken live and prepared by overnight fixation in 4% glutaraldehyde with 2.5% formaldehyde containing sodium cacodylate buffer pH 6.5, dehydrated as above, and scanned with the following setting parameters: isotropic voxel size = 0.52μm per pixel; source voltage = 47KV, source current = 51μA, image rotation step = 0.2°, 360° of rotation scan, and no filter.

For primary reconstructions and the cleaning process to compile the datasets of cross-sectional images (slices), we used the Skyscan (www.skyscan.be) software (NRecon, DataViewer, CTAnalyser). We reconstructed, re-oriented, and eliminated noise of the images as previously described [42]. Volume renderings of the images were made using FEI’s Amira software v.6.4, (using the built-in “volenRed.col” color filter) [43], except for Fig 7a, which was made with Skyscan’s software CTVox (colors were applied by varying the color-transfer function curves, in conjunction with the lighting and shading options). For a more detailed explanation of the process, see the previous paper [42].

The images of the aedeagus visualized by microtomography were compared with those made after routine light-microscope slide preparation, using one aedeagus dissected from a male terminalia mounted on a slide in Hoyer’s liquid with a cover slip (Fig 7g).

In accordance with the micro-CT results (as in the figures), the standard anatomical position is used to describe structures.

## Results

In the specimens studied, the reproductive system appears voluminous, occupying more than 50% of the abdominal volume (Fig 1). It consists of two lateral testes, appearing to have a single spindle-shaped structure narrow and elongated at both the fore and hind ends, appearing as narrow lobes (Figs 2, 3 and 5a). A vas deferens extends from the medial zone to the posterior ventral surface of the seminal vesicle (Figs 3b, 3c and 5a). Seminal vesicles appear globose, close to each other and, together with the long, ovoid accessory glands, connect to the sperm pump through short ducts (Figs 1a–1d, 3, 4 and 5a–5f).

Each testis has an anterior (frontal) and posterior (hind) lobe (Figs 2, 3a and 3c). In section, the spermatocyte cysts are visible, clearly separated by dorso-ventral walls forming a single row (Figs 3, 4a and 5f). In the middle zone appear spermatocyte cysts containing spermatozoa in their final developmental stages (Figs 3b, 3c and 4b). Vasa deferentia appear as long narrow ducts (ca. 20 μm in diameter) (Figs 2, 3b, 3c and 5a).

Two globose seminal vesicles touch each other but remain discrete (no interconnection is visible; Figs 1–3 and 5), and from the ventral-hind surface of each a vas deferens extends from the corresponding testis (Figs 3b, 3c and 5a). From the hind ventral part stems a short wide duct (ca. 50 μm in diameter) that comes together with the accessory gland duct and connects to the sperm pump (Figs 2d, 3, 4c and 5b–5f).

The accessory glands, situated lateroventrally to the sperm pump (Figs 1, 2a–2c, 3, 4a, 5f and 6d), have external and internal walls (Figs 3, 4a, 5c–5f and 6d). Inside, dense structures are visible (Figs 3b, 4a, 4c and 5f) with the size and shape of the spermatozoa described for *D*. *citri* [12]. From the inner hind dorsal end of each accessory gland extends a short duct, ovoidal in section (ca. 23 x 40 μm; Figs 2a–2d and 5b–5e).

Known classically as the sperm pump (Figs 1, 2a–2d, 3a, 3b, 4c, 5a, 5b and 5f–5n), this organ is composed of a cylindrical fore part and a long-narrow conical hind part that connects with the sperm duct. The cylindrical part consists of an anterior sclerotized, slightly oval, concave plate (ca. 63 x 87 μm) with two middle foramina separated by a dorso-ventrally aligned ridge (Fig 5j–5l). On each side of the ridge are the connections of both seminal vesicles, and accessory-gland ducts. A conical structure is connected to the fore plate through a series of longitudinal muscles appearing as parallel bands, forming a pumping chamber (ca. 50 μm in length; Figs 1a–1d, 3a, 3b, 5b, 5f–5i, 5k and 6d). The conical part contains a long piston structure fixed to the anterior part by a muscle, appearing as an attachment filament (Figs 3b, 5f and 5h). This connects the fore tip of the piston at the center of the longitudinal ridge in the inner-posterior central surface of the concave plate (Fig 5m).

A narrow ejaculatory duct (ca. 15 μm in diameter) extends from the posterior tip of the sperm pump (Figs 1, 2a–2d, 3b, 5b, 5f and 6d), connected to the aedeagus and passing through it (Fig 7f) by a foramen situated at its basal fore plate (Fig 8b and 8k).

The external genitalia (abdominal terminalia) consist of a proctiger plate (also called as anal tube [44]) with the anus opening on the dorsal tip, a subgenital plate, and two parameres (also called lateral plates [44]), acting as forceps or gonostyles (Figs 1, 3, 6 and 7a–7d). Each has a small pointed end, and numerous trichoid sensillae along the inner margins (Fig 6b–6d). Dorsally and externally the elbow joint of the first and second segment of the aedeagus is visible (Fig 6c). The internal musculature are pictured in Figs 3, 6d and 7a–7d.

The internal genitalia (Figs 1, 3a, 3b, 5f, 6d, 7 and 8), consist of a three segmented aedeagus with two long initial segments articulated by a dorsal joint, and an enlarged 3^rd^ distal-end segment, convex on the inner side and flattened on the external side, with a concavity, forming at margins two ridges that are Y shaped (Fig 8b and 8d–8f), and with a rounded tip pierced by two small apical foramina (Fig 8f). The 1^st^ segment is hook-shaped, with a rectangular (longer than wider) basal fore plate, which is basally pierced by the foramen of the ejaculatory duct. This has two antero-posterior long lateral basal apophyses, two short postero-latero-basal apophyses, and two short antero-latero-basal apophyses. The margin behind the 1^st^ segment is slightly concave on the distal third. Internally, in a resting position, the 1^st^ segment of the aeadeagus appears parallel to the paramers, while the 2^nd^ segment is parallel to dorsal edge of the proctiger and runs from a dorsal position pointing ventralwards to join the 3^rd^ segment. Laterally a conspicuous notch appears in the anterior edge, where the 2^nd^ and 3^rd^ segment join (Figs 7a, 7d–7g, 8c and 8g).

Different animated perspective views can be visualised as supporting information video files (S1 and S2 Videos).

## Discussion

Głowacka *et al*. [15] reported that the testes of *Diaphorina citri* have spermatocyte cysts arranged in one row, in agreement with our observations (Figs 3, 4 and 5f). Moreover, according to these authors each testis has two follicles, while for *Bactericera albiventris* they described and figured an almost complete fusion (but with a slight vestigial separation) of follicles. At first sight, each testis of the ACP appears to consist of a spindle-shaped single lobe. However, a detailed examination clearly reveals narrow lobes at both ends of each testis, and in sagittal sections these unequivocally correspond to the germinal zone of testes (germarium). Thus, what appears to be a single structure, actually consists of two follicles, fused into a single external structure, with a common vas deferens. This agrees with the view in sagittal sections. From each germarium, towards the vas deferens connection, spermatozoa inside spermatocyte cysts are formed and progressively mature. Spermatids/immature spermatozoa are clearly visible in the middle part of each testis (Figs 3c, 4a and 4b), similar to the description of *Aphalara polygoni* by Głowacka *et al*. [15].

Although it may seem strange to find spermatozoa in the accessory glands, the seminal vesicles and accessory gland ducts connect to the sperm pump very close together, in a small area of the concave plate, just before the seminal pump chamber (Figs 2b, 2d and 5a), so that spermatozoa could easily reach the accessory glands.

The structure classically interpreted as a sperm pump has received different names. Witlaczil [45] called it "*kolbenförmiges Organ*" (literally, “piston-shaped organ”), but it was not until Saunders [8] that the function of this structure was accurately interpreted and this researcher referred to it as an "ejaculatory pump", as did Brittain [6]. While Qadri [46] called it a “seminal pump”, Schlee used the term “sperm pump” [39] and so did Prophetou [35]. We adopt the term “sperm pump” as the most accurate name because it refers to its apparent function. The detailed structure revealed by micro-CT appears to have all the impellent suction elements of such a pump. The 3D examination provides new information to explain how the pumping functions to avoid sperm reflux. Thus, by the contraction of the longitudinal muscles surrounding the cylindrical pumping chamber (Fig 5g, 5m and 5n), the sperm would be pressed out of the chamber and into the ejaculatory duct. Simultaneously, the piston’s muscle (attached between the fore tip of the piston and the internal central surface of the inner-posterior side of the concave plate) would contract, preventing sperm reflux. Thus, the dorso-ventral fore ridge of the concave plate apparently reinforces the plate against bending during the muscle contraction.

The observed micro-CT sperm pump structure matches prior descriptions for other closely related Psyllinae species [9,39,47] as well as what Matsuda summarized [48]. Thus the new visualization improves on the previous drawing of the ACP sperm pump by Mathur [49].

The genus *Diaphorina* has a characteristic 3-segmented aedeagus. In papers, the male genitalia of *D*. *citri* are depicted with a “spoon”-shaped, dilated 3^rd^ segment, (Mathur [49], Burckhardt [50,51]), In light-microscope slides the 3^rd^ segment looks more enlarged, and spoon shaped (Fig 7g) than in a previous micro-CT study [7], or in the present study (Figs 7 and 8). This is because in light-microscope slide the 3rd segment results compressed under the cover slip and therefore deformed. In fact, Ammar *et al*. [44] published a picture from a light microscope slide of the male terminalia where the tip of the aedeagus appears unusually enlarged and spoon shaped (as in Fig 7g), but in a SEM image (where the genitalia were not compressed), the 3^rd^ segment has a shape similar to that in our micro-CT volume rendering reconstructions. The most precise anatomical descriptions of the genitalia and aedeagus of a psyllid species has been published for *Psylla crataegi* by Zucht [9], and later included in classic works, such as the one by Bitsch [10], and the one for *P*. *mali* by Muir [11] where (after microscopic slide preparation, and threafter compression) the basal fore plate (called the “basal plate”) and the long basal apophyses (called the “basal prolongations”) were depicted. However, no observations have been made on the details described above and summarised in Fig 8, and no similar study has described and/or depicted psyllids, including *D*. *citri*.

During dissection, certain animal organs such as testes, which are tethered, can spin on the tether and change their position when viewed in the open. However, inside the animal they maintain a fixed position. In fact, by micro-CT, we studied several specimens and in all of them the structures remained in similar positions, as described.

Micro-CT volume rendering images offer quality comparable to low-magnification scanning (SEM), with the additional advantage that viewing is possible from any angle or perspective, in a way not possible with electron microscopy. The rapid advance on micro-CT and nano-CT technology, with constant magnification and resolution improvements, are approaching the power of electron microscopy, representing a promising future for new discoveries.

## Supporting information

S1 Video Video of the ACP male reproductive system. Spinning animation of a micro-CT volume rendering reconstruction of the male reproductive structures, permitting to observe them from different angles. Components (testes, accessory glands, seminal vesicles, sperm pump, ejaculatory duct and aedeagus) are dissembled, rotated and assembled to their actual anatomical position.
(MP4)

S2 Video Video with cut views of the ACP male reproductive system. Spinning animation of a micro-CT volume rendering reconstruction of the male reproductive structures, with progressive sagittal and transversal cut views.
(MP4)
